# Construction of nanodelivery system based on the interaction mechanism between ultrasound–treated soybean whey protein and quercetin: structure, physicochemical stability and bioaccessibility

**DOI:** 10.1016/j.ultsonch.2024.107195

**Published:** 2024-12-10

**Authors:** Xinru Cao, Jia Cao, Tianhe Xu, Lexi Zheng, Jingyi Dai, Xiaokun Zhang, Tian Tian, Kunyu Ren, Xiaohong Tong, Huan Wang, Lianzhou Jiang

**Affiliations:** a College of Food Science, Northeast Agricultural University, Harbin 150030, China; b College of Agricultural, Northeast Agricultural University, Harbin 150030, China; c School of Food Science and Engineering, Hainan University, Haikou 570228, China

**Keywords:** Soybean whey protein, Quercetin, Ultrasound, Nanoparticles, Structure

## Abstract

In this study, soybean whey protein (SWP) nanodelivery system was constructed through ultrasound treatment and quercetin (Que) modification. The effect of ultrasound power on the interaction mode between SWP and Que, and the formation and stability of SWP–Que nanodelivery system were investigated. Optimal ultrasound treatment (300–500 W) produced SWP–Que nanoparticles with smaller particle size, higher ζ–potential values, and more uniform dispersion. Fluorescence spectroscopy and FTIR analyses revealed that SWP primarily binds to Que through hydrophobic interactions. Ultrasound treatment induced the unfolding of the SWP structure, thereby increasing its binding affinity to Que. After 400 W sonication, the encapsulation efficiency can reach 95.63 ± 0.60 %. The SWP–Que nanoparticles protected Que from degradation under environmental stresses (heat, UV, and storage) and improved its bioaccessibility during digestion as the ultrasonic power of 400 W. This study highlights the potential of ultrasound–modified SWP nanoparticles for effective nutrient delivery.

## Graphical abstract

**Figure:**
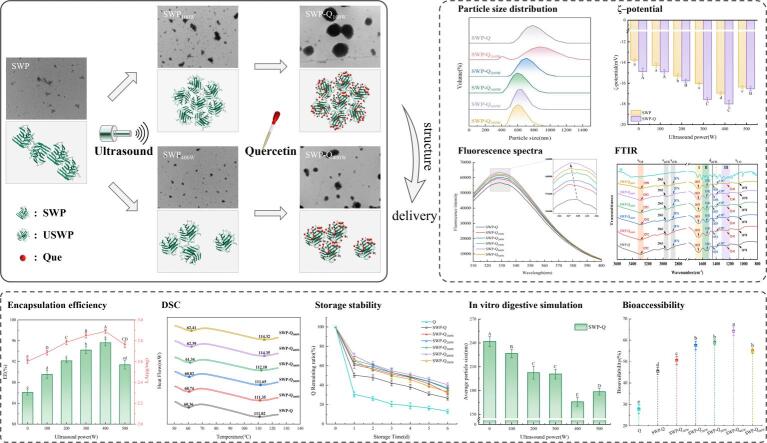

## 1 Introduction

Soybean whey protein (SWP) is a by–product of tofu and soybean protein production in food processing. It has several advantages, including a large quantity, a high amino acid fraction and excellent physiological activity. It has applications not only as a nutritional and functional ingredient in various food formulations, but also in the delivery carrier of active substances [1]. Proteins can spontaneously bind to polyphenols through non–covalent interactions such as hydrophobic interactions, thereby protecting the polyphenols and reducing their oxidation and degradation under various environmental stresses [2]. The SWP–gum arabic–curcumin emulsion delivery system constructed by Cao et al. [3] can provide effective protection for curcumin when external conditions change. Furthermore, Xiang et al. [4] successfully prepared stable nanoparticles using pyridine–grafted poly (hydroxyethyl methacrylate) and SWP by non–covalent self–assembly, which exhibited excellent photostability and effectively prevented the decomposition of Bingqingxiao under UV irradiation.

In order to modify the structure of natural proteins and enhance their suitability for production, various technological approaches have been employed. Among these, ultrasound technology represents a novel non–thermal processing technology that offers the benefits of safety, environmental protection and economy. In aqueous extraction media, ultrasound generates high shear energy and macroscopic turbulence by forming cavitation bubbles that collapse violently under high pressure [5]. The cavitation effect generated during the ultrasonic processing can disrupt non–covalent bonds between protein molecules or within molecules, such as hydrogen bonds and hydrophobic interactions. On the one hand, protein molecules partially unfold, exposing buried hydrophobic regions within the protein to the surrounding medium [6]. The increased hydrophobic groups lead to a significant reduction in the thermodynamic energy barrier, thereby increasing molecular collision rates [7]. On the other hand, the tight structure of protein aggregates in the solution is disrupted, resulting in increased surface area due to their dispersion. This provides more possibilities for proteins to interact with water or biologically active substances (e.g., polyphenols) [8], [9]. For instance, Meng et al. [10] found that soybean isolate protein (SPI) treated with 20/40 kHz sonication showed increased exposure to hydrophobic regions and enhanced binding to curcumin.

Quercetin (Que) is a flavonoid polyphenol substance abundant in vegetables (e.g., onions, broccoli), fruits (e.g., apples, blueberries), grains, and herbs. It has been demonstrated to possess antioxidant, anticancer, and anti–inflammatory properties [11]. However, the crystalline nature and low water solubility of Que result in poor bioavailability within the body following consumption. Furthermore, Que is chemically unstable when exposed to environmental factors such as oxygen, light and high temperatures, making its direct incorporation into foods and liquid functional foods a challenging process [12]. Nanoparticles are dispersed systems with particle sizes between 1 and 1,000 nm, where the matrix exists in the form of encapsulation, adsorption and dispersion. Studies have been shown that encapsulating plant polyphenols using delivery systems of edible nanoparticles improves their dispersion, stability and bioavailability [2], [13]. Que can bind to a variety of food–borne proteins and is often protected by being encapsulated in protein nanoparticles [14]. In a study conducted by Wang et al. [15], the potential of SPI particles as a protective carrier for Que was investigated. The findings revealed that SPI particles exhibited superior performance to natural SPI in enhancing the stability and free radical scavenging activity of Que.

At present, there is a paucity of research examining SWP–polyphenol complexes or the utilisation of SWP as a polyphenol delivery vehicle. The effect of SWP nanoparticles prepared by ultrasound treatment on the stability and digestive properties of polyphenols remains an area for further elucidation. In this study, SWP and SWP–Que self–assembled nanoparticles were prepared by ultrasound treatment of SWP at different power levels. The structure of nanoparticles, their stability under different environmental pressures and digestion characteristics were investigated. The aim was to investigate the potential of ultrasound treatment in facilitating SWP–Que interaction, and in SWP protection and delivery of Que, with the objective of enhancing the bioavailability and application potential of hydrophobic nutraceuticals to increase their potential health benefits.

## 2 Materials and methods

### 2.1 Materials

The defatted soybean flakes were generously provided by the Yihai Kerry Cereals, Oils and Foodstuffs Co., Ltd. (Qinhuangdao, China). Que (purity ≥ 97 %) was purchased from Yuanye Bio–Technology Co., Ltd. (Shanghai, China). Pepsin (≥2,500 U/mg) and trypsin (3,000 U/mg) were procured from Sigma–Aldrich Co., Ltd. (Shanghai, China). All other chemicals and solvents used were of analytical grade.

### 2.2 Preparation of SWP

The extraction method for SWP was based on the method reported by Cao et al. [16], with minor modifications. Defatted soybean powder was uniformly dispersed in distilled water (1:10, w/v), and the pH of the dispersion was stabilised at 8.0 with stirring for 2 h. The dispersion was centrifuged (8,000 rpm, 30 min) and the supernatant was extracted. The pH was adjusted to 4.5, stirred at room temperature for 30 min, and then centrifuged (8,000 rpm, 20 min) to collect the supernatant. The supernatant is the crude soybean whey solution. Add ammonium sulfate to the crude soybean whey protein solution to 80 % saturation and then centrifuged (8,000 rpm, 30 min). The centrifuged precipitate was redissolved in distilled water and dialysed (MWCO 3,500 Da, 4 °C) for 48 h with regular water changes. The dialysed solution was lyophilised for 24 h to obtain SWP, and the protein content of the lyophilised SWP was determined to be 90.45 ± 0.75 % using a Dumas nitrogen tester (NDA702, VELP, Italy).

### 2.3 Preparation of SWP–Que nanoparticles

A 1.0 % (w/v) solution of SWP was prepared and stirred for 2 h followed by hydration at 4 °C overnight. After the solution returned to room temperature, the pH was adjusted to 7.0 to obtain the initial SWP solution. The SWP solution was sonicated using an ultrasound cell crusher (Scientz–IID, NingBo Scientz Biotechnology Co. Ltd., Ningbo, China) with different powers (0, 100, 200, 300, 400, 500 W) for a total ultrasound treatment time of 10 min (5 s of treatment and 5 s of interval). The SWP solutions obtained from the ultrasound treatment were named SWP, SWP_100W_, SWP_200W_, SWP_300W_, SWP_400W_, SWP_500W_.

Firstly, a 10 mM Que ethanol solution was prepared as Que stock solution. A SWP–Que mixed solution was prepared by mixing the unsonicated or sonicated SWP solution with the Que stock solution. The final sample contained 10 mg/mL of SWP along with 1 mM of Que. The SWP–Que composite nanoparticle solution was prepared by stirring the mixed solution away from light for 2 h [17]. The obtained SWP–Que hybrid solutions were named SWP–Q, SWP–Q_100W_, SWP–Q_200W_, SWP–Q_300W_, SWP–Q_400W_, SWP–Q_500W_.

### 2.4 Particle size and ζ–potential

Samples were diluted 50–fold and Zetasizer Nano ZSP (Malvern Panalytical, UK) was used to determine particle size and ζ–potential. The refractive indexes of the protein and water were set to 1.46 and 1.33, respectively. All sample solutions were measured at 20 °C [18].

### 2.5 Fourier transform infrared spectroscopy (FTIR)

The FTIR spectra of lyophilised samples were recorded in the wave number range of 4,000 to 400 cm^−1^ using a FTIR spectrometer (Thermo Nicolet Ltd, USA). The secondary structure of the samples within the amide I region (1,700–1,600 cm^−1^) was analysed utilising the PeakFit version 4.12 software (Inc., Chicago, IL, USA) [19].

### 2.6 Fluorescence spectra

Prior to measuring the fluorescence spectra, the samples were subjected to a 100–fold dilution. The fluorescence spectra were then recorded at an excitation wavelength of 280 nm using a fluorescence spectrophotometer (F–4500, Hitachi, Japan) [20].

### 2.7 Surface hydrophobicity (H_0_)

The samples were diluted to achieve a protein concentration of 0.001–0.2 mg/mL. The sample was mixed with 1–anilino–8–naphthalene–sulfonate (ANS) solution (10 mM) at 100:1 v/v ratio, vortexed and shaken and allowed to react for 15 min. H_0_ was determined and calculated according to Tian et al. [21].

### 2.8 Solubility

The solubility of the proteins in the samples was determined through the utilization of the bicinchoninic acid (BCA) method. The sample is diluted and centrifuged at 10,000 rpm for 10 min. The solubility was calculated by employing a ratio of the concentrations calculated in the supernatant to the total protein concentrations [22].

### 2.9 Transmission electron microscopy (TEM)

Dilute the fresh sample solution to 0.1 mg/mL and take one drop into the carbon film copper grid. After being placed under an incandescent lamp for 1 min, the excess sample was gently removed with filter paper. Subsequently, an appropriate amount of phosphotungstic acid (1 %, w/v) was applied to copper mesh, and the excess stain was removed by aspiration after 2 min of staining. After drying, the micmorphology of the samples was observed using a TEM (HT 7800, Hitachi High–Technologies Corp, Tokyo, Japan) [23].

### 2.10 Encapsulation efficiency (EE) and loading amount (LA)

The EE and LA were conducted in accordance with the methodology outlined in the published protocol [24]. The EE of Que encapsulated by SWP can be calculated by the following formula:

(1) $$\mathrm{EE}\;(\%)\;=\;\frac{\mathrm{total}\;\mathrm{amount}\;\mathrm{of}\;\mathrm{Que}\;-\mathrm{amount}\;\mathrm{of}\;\mathrm{free}\;\mathrm{Que}}{\mathrm{total}\;\mathrm{amount}\;\mathrm{of}\;\mathrm{Que}}\times 100$$

where, quantity of Que present in the precipitate following centrifugation was determined to be the free Que content. To this end, the precipitate was completely solubilized using 95 % ethanol (w/w) and then centrifuged (10,000 rpm, 15 min). The concentration of Que in the supernatant was determined by quantifying the amount of Que in accordance with the standard curve at a wavelength of 374 nm. The LA of Que encapsulated by SWP can be calculated using the following formula:

(2) $$\mathrm{LA}\;\left(\mu g/\mathrm{mg}\right)=\frac{\mathrm{total}\;amount\;of\;Que\;-\;amount\;of\;free\;Que}{\mathrm{total}\;amount\;of\;SWP}$$

### 2.11 Differential scanning calorimetry (DSC)

The methodology described by Tian et al. [25] was modified slightly. Approximately 7 mg of samples were placed in aluminium pans and assayed using DSC (Mettler Toledo Corp., Zurich, Switzerland). The samples were subjected to a heating process, increasing the temperature from 20 °C to 130 °C at a rate of 10 °C/min.

### 2.12 Antioxidant capacity

The antioxidant activity of the samples was determined by calculating the DPPH and ABTS^+^ radical scavenging rates pursuant to the previously established methodology [26], with minor modifications.

#### 2.12.1 DPPH radical scavenging rate

A 0.1 mM ethanol solution of DPPH was configured and combined in equal volume with the sample (2 mg/mL). The mixed solution was transferred to a dark environment for 30 min, after which the absorbance was determined using a ultraviolet spectrophotometer. The DPPH radical scavenging rate (%) was calculated as follows:

(3) $$\mathrm{DPPH}\;\mathrm{radical}\;\mathrm{scavenging}\;\mathrm{rate}\;(\%)\;=\frac{A_{0}\;\mathrm{- A}}{A_{0}}\times 100$$

where, A and A_0_ are the absorbance of the sample and the DPPH ethanol solution, respectively, at 517 nm.

#### 2.12.2 ABTS^+^ radical scavenging rate

A solution of 7.4 mM aqueous ABTS^+^ was mixed with 2.6 mM of aqueous potassium persulfate in equal proportions and reacted for 16 h away from light. Subsequently, the working solution were combined with the sample solution in a 4:1 v/v ratio. Following a 6 min incubation period, the sample absorbance was quantified at 734 nm. The ABTS^+^ radical scavenging rate (%) was then calculated using the following equation:

(4) $${\mathrm{ABTS}}^{+}\;\mathrm{radical}\;\mathrm{scavenging}\;\mathrm{rate}\;(\%)\;=\frac{A_{0}\;\mathrm{- A}}{A_{0}}\times 100$$

where, A and A_0_ are the absorbance at 734 nm for the reaction of the sample and ethanol with the ABTS^+^ radical working solution, respectively.

### 2.13 Evaluation of stability

#### 2.13.1 Thermal stability

The thermal stability of Que contained in SWP–Que nanoparticles was analysed in reference to Cui et al. [27]. The SWP–Que solution was subjected to a 10 min boiling process in a water bath, after which the Que content was quantified in accordance with the methodology delineated in Section 2.10. To ascertain the retention rate of Que, the following formula was utilized:

(5) $$\mathrm{Retention}\;\mathrm{rate}\;(\%)\;=\;\frac{C}{C_{0}}\times 100$$

where C_0_ and C are the amount of Que in the system before and after heating, respectively.

#### 2.13.2 UV stability

The SWP–Que solution should be exposed to UV light for a period of 150 min, with a wavelength of 340 nm and an intensity of 10 W. A portion of the sample was removed at regular intervals and the amount of Que in the sample during UV treatment was determined in accordance with the methodology outlined in Section 2.10. To ascertain the retention rate of Que, the following formula was utilized:

(6) $$\mathrm{Retention}\;\mathrm{rate}\;(\%)\;=\;\frac{C_{t}}{C_{0}}\times 100$$

where C_0_ and C_t_ are the Que content in the system at treatment times 0 and t, respectively [3].

#### 2.13.3 Storage stability

The SWP–Que solution was stored at 4 °C for 6 d to assess the storage stability of Que within the system. During the storage period, a portion of the sample was taken out at the same time each day to determine the Que content of the sample. To ascertain the retention rate of Que, the following formula was utilized:

(7) $$\mathrm{Retention}\;\mathrm{rate}\;(\%)\;=\;\frac{C_{t}}{C_{0}}\times 100$$

where C_0_ and C_t_ are the Que content in the system at treatment times 0 and t d, respectively [17], [28].

### 2.14 Digestive properties of SWP and SWP–Que nanoparticles

#### 2.14.1 In vitro digestive simulation

The in vitro digestion study builds upon a previously conducted study, incorporating certain modifications [29]. Briefly, the preparation of the samples was conducted and then blended with an equal volume of simulated gastric fluid of pH 2. The solution was subjected to a two–hour gastric digestion simulation in a water bath shaker maintained at 37 °C. Once the process was complete, the pH of the gastric digestion products was adjusted to 7.0 solution. Subsequently, the gastric digestion products were mixed in equal volumes with simulated intestinal fluid. shaking at 37 °C for 2 h to simulate intestinal digestion. Upon completion of digestion, the digested solution was transferred to ice water bath for enzyme inactivation.

#### 2.14.2 Determination of particle size

The methodology employed was consistent with that outlined in Section 2.4 of the study.

#### 2.14.3 Bioaccessibility determination of Que

The methodology employed was consistent with that outlined in Section 2.10, and the bioaccessibility of the compounds under investigation was calculated using the following equation:

(8) $$\mathrm{Bioaccessibility}\;(\%)\;=\;\frac{C}{C_{0}}\times 100$$

where, C_0_ represents the quantity of content in the complexes prior to digestion, whereas C denotes the quantity of content in the supernatant subsequent to complexes digestion.

### 2.15 Statistical analysis

The experiments were performed a minimum of three times, and the resulting data were presented as mean ± standard deviation (SD). We determined the significance of ANOVA was used to analysis of variance by applying SPSS 23.0 software. The level of statistical significance was set at *p* < 0.05, which is considered to be statistically significant. The graphs were generated using Origin 2020 software.

## 3 Results and discussion

### 3.1 Particle size analysis

**Fig. 1:**
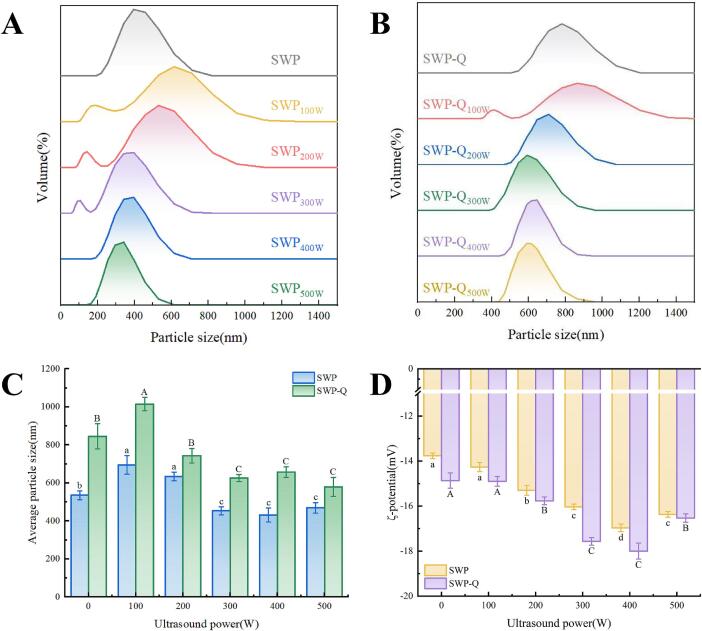
Particle size distribution (A, B), mean particle size (C) and ζ–potential (D) of SWP and SWP–Que nanoparticles at different ultrasound powers. Different lowercase letters and uppercase letters indicate significant differences in means within the same parameter group (*p* < 0.05).

The average particle size and particle size distribution of SWP and SWP–Que nanoparticles under different ultrasound powers are shown in Fig. 1**A–C.** It was observed that the average particle size of natural SWP was small with a unimodal distribution. The particle size of SWP–Que was larger than that of protein nanoparticles (*p* < 0.05). This indicates that Que forms larger structures or aggregates through certain interactions with SWP [30]. The average particle size of SWP–Que nanoparticles was increased from 845.43 ± 65.62 nm to 1,015.50 ± 34.66 nm after 100 W ultrasound treatment, and the particle size distribution curve shifted towards larger particles. The observed result may be attributed to the low–power ultrasound enhancing the velocity of collisions and particle aggregation in the presence of turbulence and microfluidic effects. This consequently gives rise to the formation of soluble aggregates and an increase in particle size [31]. The driving force for this aggregate formation may involve electrostatic and hydrophobic interactions [32]. Conversely, an increase in ultrasound power (300–500 W) was observed to result in a decrease in the average particle size of the SWP–Que nanoparticles decreased (*p* < 0.05). This is because the increase of ultrasound power, unstable aggregates were broken down into smaller soluble protein aggregates due to the intense turbulence and cavitation [33].

### 3.2 ζ–potential analysis

The ζ–potential can provide insight into the stability of the system to a certain extent. In general, a ζ–potential threshold close to ± 30 mA is considered to indicate high system stability. The closer the ζ–potential value of the system is to the threshold, the lower the tendency for aggregation, indicating better stability [34]. As illustrated in Fig. 1**D**, all samples exhibited a negative ζ–potential at pH 7.0 due to the electronegativity of SWP in neutral dispersion. The net potential value of the natural SWP was 13.77 ± 0.12 mV, and both the ultrasound treatment and the addition of Que exhibited an increase in the net ζ–potential value. This indicates that there has been an enhancement in the electrostatic repulsion between the nanoparticles. The net ζ–potential of the composite nanoparticles exhibited an incremental increase, reaching a value of 18.12 ± 0.36 mV with an augmentation in the treatment power from 0 W to 400 W. Ultrasound treatment opened up the initially dense protein structure, exposing polar groups initially embedded within the protein were exposed to the surface of the protein particle. This, in turn, resulted in a notable increase in the absolute value of the ζ–potential [35]. Whereas, the net ζ–potential of SWP–Q_500W_ decreased slightly (16.53 ± 0.19 mV). The formation of soluble aggregates may be responsible for this phenomenon, as it results in a reduction of repulsive forces and surface charge [23].

### 3.3 FTIR analysis

**Fig. 2:**
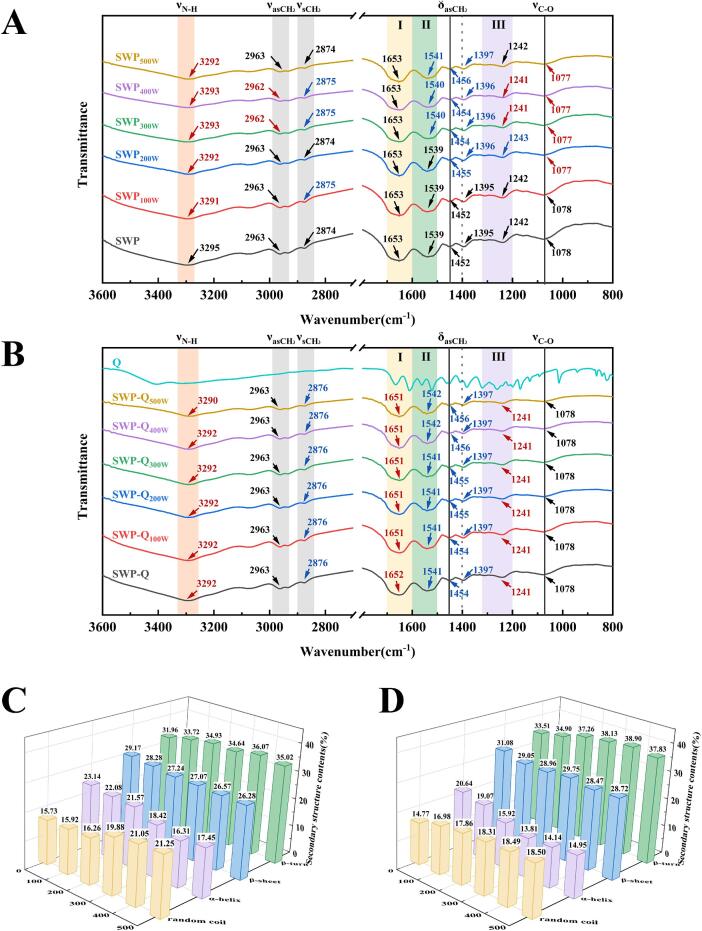
(A, B) FTIR of Que, SWP, and SWP–Que nanoparticles; (C) Secondary structure content of SWP, SWP–Que nanoparticles.

Insights into the molecular mechanism of SWP–Que interactions were gained by FTIR analysis. As demonstrated in Fig. 2**A–B**, ultrasound power and the addition of Que influenced the characteristic peaks of SWP. In the SWP–Que nanoparticles, the characteristic peaks belonging to the Que disappeared, indicating that the Que was successfully encapsulated in the nanoparticles [36]. The absence of new absorption bands following the binding of Que to SWP indicates that, in these matrices, only a physical binding occurred. After ultrasound treatment and addition of Que, the characteristic peak associated with SWP, occurring at 3,295 cm^−1^, underwent a red–shift to varying degrees as an indirect consequence of intermolecular hydrogen bonding and the presence of O–H and N–H stretching vibrations. This indicates the presence of hydrogen bonding between SWP and Que [37]. In addition, compared to SWP, SWP–Que exhibited red–shifted peaks in the amide I band and blue–shifted peaks in the amide II band. These peak positions changes were associated with hydrophobic interactions between SWP and Que [24]. The findings imply that the application of ultrasound and the interplay between SWP and Que result in the extension and curvature of a multitude of functional groups, which may potentially engender alterations in protein configuration.

In order to characterise the impact of ultrasound treatment on the secondary structure of SWP, the peaks were fitted to the amide I band (1,600–1,700 cm^−1^) of FTIR (Fig. 2**C–D**). With the increase in ultrasound power, the relative content of α**–**helix and β**–**sheet structures decreases, while the relative content of β**–**turn and random coil structures increases. This indicates that ultrasound treatment led to an increase in the disorder of SWP structure. Furthermore, the addition of Que also affected the secondary structure content of SWP. The relative contents of β–sheet and β–turn in composite nanoparticles increased, while the content of random coils decreased. Among them, under the conditions of 400 W ultrasound treatment, the secondary structure change of composite nanoparticles is most significant (*p* < 0.05), with the relative content of β–turn increasing to 38.90 ± 0.84 % and the content of α–helix decreasing to 14.14 ± 0.69 %. Ultrasound treatment causes the structure of SWP to become more extended, thereby exposing hydrophobic and polar groups within the peptide chain to the surface, increasing the interaction between SWP and quercetin [38]. This outcome is also aligned with the findings of Ji et al. [17] regarding the interaction of sonication on Que and whey protein concentrate.

### 3.4 Fluorescence spectroscopy and surface hydrophobicity analysis

**Fig. 3:**
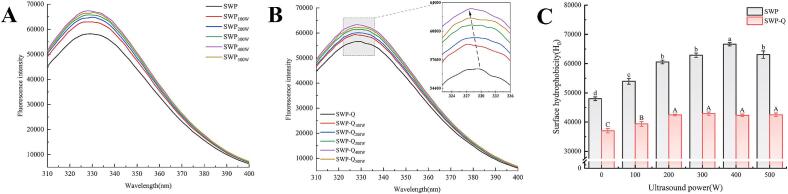
Fluorescence spectra (A, B) and surface hydrophobicity (C) of SWP and SWP–Que nanoparticles at different ultrasound powers. Means with different lowercase and uppercase letters were statistically significantly different from each other (*p* < 0.05).

Fluorescence spectra reflect the properties of aromatic amino acids in proteins, thereby characterizing their tertiary structure [39]. As shown in Fig. 3**A–B**, ultrasound treatment enhanced the fluorescence intensity of SWP and increased with the increase in ultrasound power. This indicates that cavitation, microfluidics, and turbulence generated form ultrasound treatment dissociated and unfolded the SWP aggregates and exposed the hydrophobic groups to the medium [17]. However, very high intensities may lead to protein folding or aggregation, as observed with reduced fluorescence intensity at 500 W ultrasound power [40]. Furthermore, the addition of Que significantly reduced the fluorescence intensity of SWP and shifted λmax towards shorter wavelengths (blue shift). This is thought to occur by Que interacting with hydrophobic amino acid residues, making them less hydrophobic [41].

Fluorescence results primarily characterise changes in protein microenvironmental fluorophore functionalities. The H_0_ of proteins assesses the conformational change in proteins by characterising the number of hydrophobic groups on the surface of the protein molecule [42]. As shown in Fig. 3**C**, the H_0_ for SWP tended to increase and subsequently decrease with the escalation of ultrasound power, reaching a maximum value of 66,636.73 ± 549.18 at 400 W. The cavitation effect created by ultrasound weakens intermolecular forces and hydrogen bonds, which leads to the unfolding of the protein structure. This causes hydrophobic groups within the molecules to become exposed to the polar environment, increasing the H_0_ of the sample [43]. The H_0_ was significantly lower in the SWP–Que nanoparticles compared to SWP (*p* < 0.05). Of these, untreated SWP complexed with Que had the lowest H_0_ of 37,052.67 ± 37.25. The aromatic rings and hydroxyl groups within the Que structure interact with the hydrophobic regions on the SWP surface, causing a reduction in H_0_. Increasing the ultrasonic power from 0 W to 200 W resulted in an observable rise in the H_0_ of SWP–Que nanoparticles. Nevertheless, no notable distinction (*p* > 0.05) was observed in the H_0_ of the complexes when the ultrasound treatment intensity was further elevated to 500 W. This phenomenon may be explained by structural change of SWP after appropriate ultrasound treatment, which unfolds and creates more binding sites. This structural change of SWP is more favourable for Que to bind through hydrophobic interactions [44].

### 3.5 Solubility analysis

Protein solubility significantly influences its functional properties and is often related to the balance of intermolecular repulsive and attractive forces caused by changes in protein conformation [45]. Fig. 5**A** showed that the solubility of natural SWP was relatively low at 75.75 ± 0.65 % and ultrasound treatment significantly increased its solubility (*p* < 0.05). This increase is thought to be due to the ultrasound's cavitation effect, which breaks hydrogen bonds and hydrophobic interactions, transforming insoluble macromolecules into soluble protein aggregates [46]. The addition of Que during the ultrasound treatment of SWP further improved the solubility of the nanoparticles. The highest solubility of the SWP–Que nanoparticles, 85.93 ± 1.14 %, was achieved at an ultrasound treatment power of 400 W. It is plausible that this outcome is attributed to the potential of the hydroxyl group (–OH) in Que to establish hydrogen bonds with water molecules, which may enhance protein hydration and solubility [47].

### 3.6 TEM analysis

**Fig. 4:**
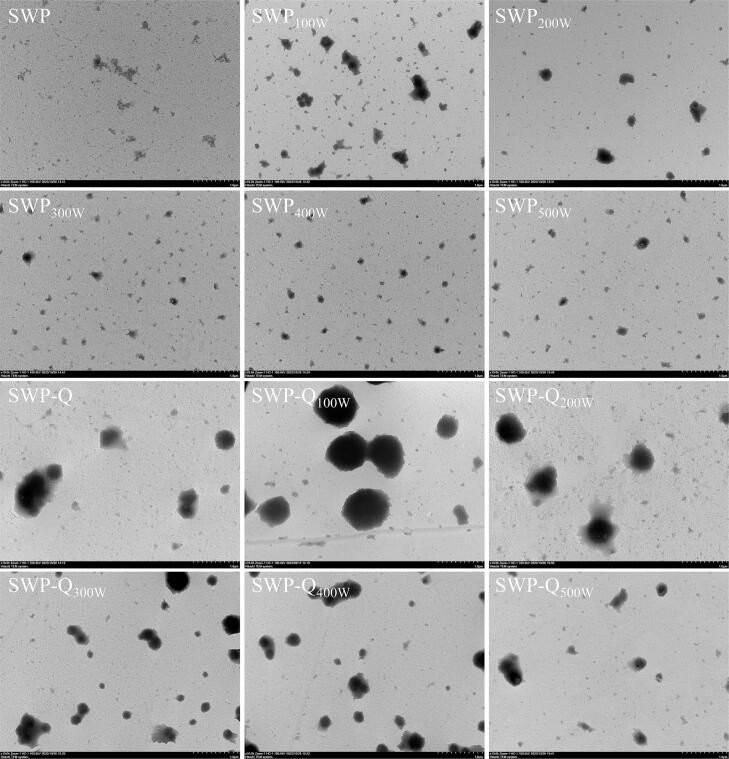
TEM images of SWP and SWP–Que nanoparticles at different ultrasound powers.

The micro morphology of SWP and SWP–Que composite nanoparticles were visualised using TEM. As illustrated in Fig. 4, the natural SWP were spherical particles with small size, but tend to aggregate into irregular flocculent structure [4]. Ultrasound treatment has caused a change in the aggregation form of proteins, resulting in the appearance of aggregates of different sizes. This may be due to the mechanical effects of ultrasound, which force the protein aggregates to have a looser structure, causing larger aggregates to disperse into smaller particles [48]. After the addition of Que, SWP and Que were successfully complexed to form nanoparticles with larger sizes. In the composite system of natural SWP and Que, many proteins remained uncomplexed with Que. The significant reduction of free SWP in the composite system after ultrasonication indicates that ultrasonication treatment is favorable for the combination of SWP with Que to form nanoparticles. Among them, under the condition of lower ultrasound power, SWP–Que nanoparticles formed large aggregates with poor dispersion. With increasing ultrasound power, the aggregation phenomenon of composite nanoparticles was significantly improved and the particle size was more uniform [49]. Especially when the ultrasound power was 400 W, a large number of evenly distributed small aggregates were formed. This matches the particle size results in Section 3.1.

### 3.7 EE and LA analysis

**Fig. 5:**
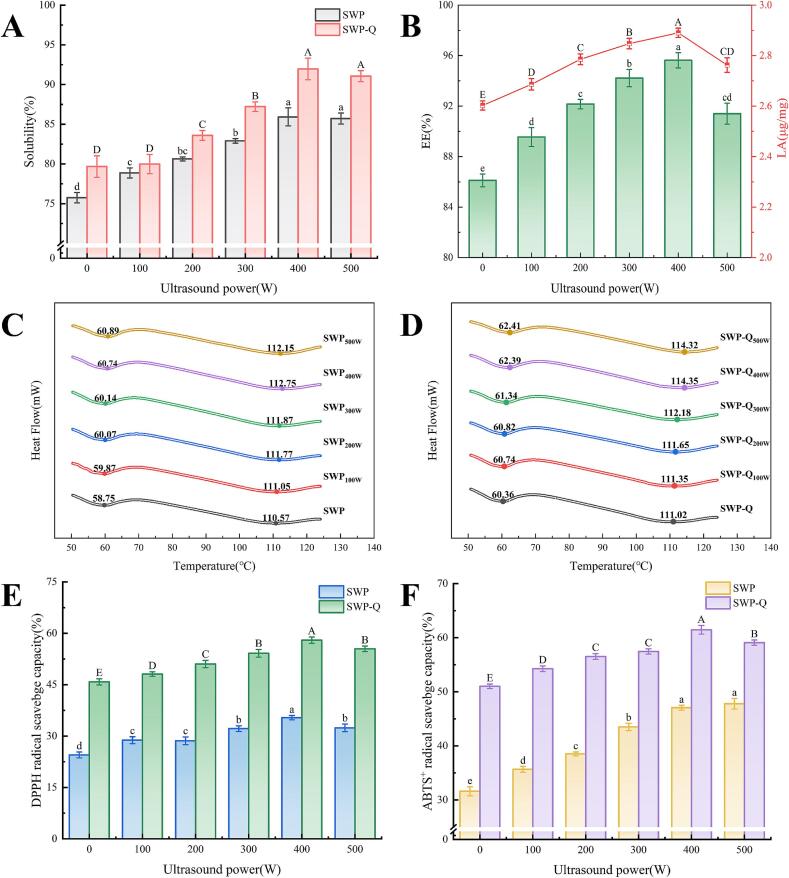
Results of solubility (A), EE, LA (B), DSC (C–D), DPPH radical scavenging capacity (E) and ABTS^+^ radical scavenging capacity (F) of SWP and SWP–Que nanoparticles. Different lowercase and uppercase letters indicate significant differences between samples (*p* < 0.05).

Fig. 5**B** shows the effects of SWP on Que EE and LA at different ultrasound power treatments. As the power of the ultrasound increased, there was an initial tendency for the EE and LA of Que by SWP to increase before subsequently decreasing. Hydrophobic polyphenols primarily interact with the hydrophobic regions of proteins. Nevertheless, the hydrophobic region of natural SWP tends to be encapsulated internally, which results in a relatively low binding rate to Que with an EE of 86.11 ± 0.50 %. The cavitation shock generated by sonication reduces the intermolecular forces and hydrogen bonding, ultimately exposing the hydrophobic groups previously hidden within the SWP molecule to the external environment as the protein unfolds. As a result, the binding of SWP to Que is enhanced, leading to an increase in EE and LA. Both EE and LA were highest at a sonication power of 400 W, 95.63 ± 0.61 % and 2.89 ± 0.02 μg/mg, respectively. Increasing the ultrasound power from 400 W to 500 W led to a reduction in EE and LA of SWP (to 91.40 ± 0.83 %), suggesting that high power sonication induces a protein aggregation reaction and buries the hydrophobic regions. This reduces the binding region with Que, resulting in lower LA and EE [6].

### 3.8 DSC analysis

DSC is an effective means of elucidating the thermodynamic properties of protein thermal transformations, including protein denaturation processes. The temperature at which denaturation occurs has been shown to be correlated with the conformational stability of proteins. It has been demonstrated that the resistance to protein unfolding increases with rising thermal denaturation temperature [29]. Fig. 5**C–D** shows that the thermal denaturation curve of SWP has two peaks of heat absorption denaturation at 58.75 °C and 110.57 °C. These correspond to the two main components of SWP: soybean trypsin inhibitor and soybean lectin, respectively [50]. Similarly, the peak shapes and trends of the DSC curves of the SWP–Que nanoparticles did not differ much from those of SWP. However, the Tm values of the composite nanoparticles were all higher than the corresponding ultrasonically treated proteins. This is due to the denser intermolecular structure and more stable conformation due to the noncovalent binding of SWP to Que. Changes in the thermal stability of proteins are usually associated with changes in the conformation of the proteins [37]. The analysis of the thermal denaturation curves indicated that the peak shape remained unaltered following the application of sonication. The Tm_1_ of composite nanoparticles showed a slight increase with increasing ultrasound power, while the Tm_2_ showed an increasing and then decreasing trend. This discrepancy is attributable to the fact that ultrasound treatment leads to incomplete or complete unfolding of proteins; which may also lead to protein repolymerization [6].

### 3.9 Antioxidant capacity analysis

The antioxidant capacity of phenolic–containing compounds serves as a pivotal indicator of their capacity to provide beneficial effects on human health [51]. The impact of sonication and addition of Que on the antioxidant capacity of nanoparticles was investigated by DPPH assay and ABTS^+^ assay [52]. As shown in Fig. 5**E–F**, the scavenging capacity of natural SWP for DPPH and ABTS^+^ radicals were weak at 24.5 ± 0.87 % and 31.6 ± 0.85 %, respectively. The SWP after ultrasound treatment exhibited higher DPPH and ABTS^+^ free radical scavenging capacity, especially at 400 W where the removal rates were 35.38 ± 0.64 % and 53.87 ± 0.83 %, respectively. The enhanced antioxidant capacity may also be attributed to ultrasound–induced protein unfolding, which exposes previously hidden antioxidant amino acid residues and side chains within the protein structure [53].

The system exhibited an enhanced capacity to scavenge free radicals with the incorporation of Que. This may be attributed to the increase in the quantity of hydroxyl groups on the surface of proteins that occurs subsequent to the addition of polyphenols [54]. Furthermore, the scavenging ability of composite nanoparticles for DPPH radicals demonstrated a gradual increase followed by a decline as the ultrasound treatment power was augmented. Upon increasing the ultrasound power from 0 to 400 W, the scavenging capacity of the SWP–Que nanoparticles for DPPH radicals exhibited a gradual increase, from 45.83 ± 0.89 % to 57.98 ± 0.93 %. When the ultrasound power continued to increase to 500 W, the antioxidant property of the SWP–Que nanoparticles slightly decreased (55.46 ± 0.74 %). Similarly, ultrasound treatment significantly increased the ABTS^+^ radical scavenging ability of SWP.

### 3.10 Thermal, UV and storage stability analysis

#### 3.10.1 Thermal stability

**Fig. 6:**
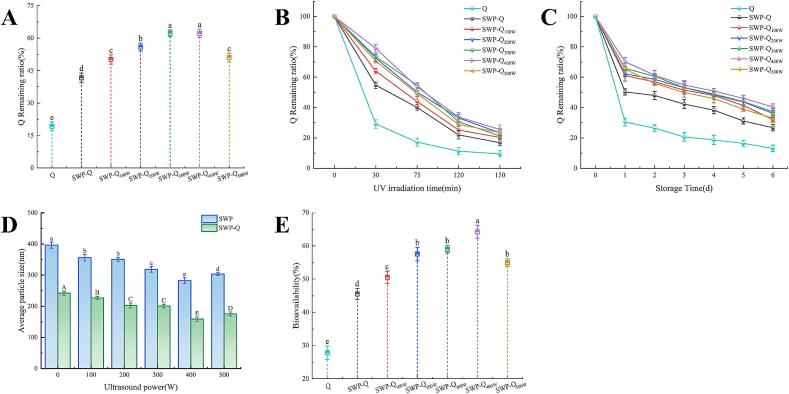
Thermal stability (A), UV stability (B), storage stability (C), particle size of digestion products (D) and Que bioavailability (E) of nanoparticles. Different lowercase and uppercase letters indicate significant differences between samples (*p* < 0.05).

Thermal stability is a process that is employed with great regularity within the context of modern food processing. To provide more effective theoretical guidance for practical applications, this experiment explored the retention rate of free Que and Que in the composite nanoparticles at 100 °C and after heat treatment for 10 min. As illustrated in Fig. 6**A**, the retention rate of free Que after heat treatment was only 19.21 ± 2.06 %, while complexing with SWP increased the retention to more than two times. This suggests that heat treatment destabilizes the system to some extent and thus accelerate the degradation of Que. The hydrophobic core region of the protein particles can serve to protect the Que in the composite nanoparticles, thereby reducing the impact of environmental stressors [55]. As the power of sonication increases, the EE of Que after heating shows a tendency to increase and then decrease. It indicates that appropriate sonication treatment to the proteins can markedly enhance the thermal stability of Que within the composite system, whereas excessively high sonication power does not consistently increase the thermal stability of Que.

#### 3.10.2 UV stability

The accelerated deterioration of Que is attributed to the disruption of the aromatic ring by UV radiation, and intense UV irradiation leads to its structural decomposition. Fig. 6**B** shows the encapsulation rate of Que at different UV exposure times. Both free or protein–bound Que degraded rapidly with increasing exposure time. Among them, Que in composite nanoparticles with SWP–Que exhibited better UV stability than free Que. After 150 min irradiated by UV light, the retention rate of Que alone was only 9.57 ± 2.02 %. With the increase of SWP power of sonication, the retention rate of Que increased first and then decreased. The SWP–Q_400W_ group had the highest retention rate of 25.52 ± 3.03 %. This may be explained by the dual protective effect of the protein, which provides both physical and chemical protection. First, SWP bound to Que can act as a shell, which serves as a physical barrier against heat and UV light. Second, SWP forms composite nanoparticles with Que through hydrophobic interactions, hydrogen bonding, and other interactions, which may play a role in stabilizing Que in the system [56].

#### 3.10.3 Storage stability

Fig. 6**C** shows the retention of free Que and Que in SWP–Que nanoparticles from 0 to 6 d. Obviously, free Que was rapidly degraded during the assay. In contrast, complexing with SWP to form SWP–Que nanoparticles effectively avoid the degradation of Que during storage. Furthermore, ultrasonically treated SWP exhibited stronger protective effect on Que. As the ultrasound treated power increases from 0 W to 400 W, the retention rate of Que during the experimental time also shows an upward trend. However, when the ultrasound power continues to increase to 500 W, the retention rate actually decreases. The retention of Que in the SWP–Q_400W_ nanoparticles being 1.51 times higher than that in SWP–Que on day 6. This may be related to the increase of hydrophobic groups on the SWP surface after ultrasound treatment [28]. This improvement may be due to the fact that ultrasonication favors the tight binding of SWP to Que, thereby improving the storage stability of Que [57].

### 3.11 In vitro digestion analysis

#### 3.11.1 Particle size analysis

In order to assess the influence of different treatments on protein digestibility, experiments were performed to simulate the in vitro gastrointestinal digestion of SWP and SWP–Que. As shown in Fig. 6**D**, the particle size of SWP only decreased from 535.37 ± 22.89 nm to 396.21 ± 9.84 nm after the simulated digestion experiment. This may be due to the trypsin inhibitor in the SWP that limit digestion. Following the application of ultrasound, a notable reduction in particle size was observed in the SWP digestion products. Concurrently, the increasing ultrasound power led to a decrease in particle size, which subsequently exhibited an increasing trend. This indicates that ultrasound treatment may facilitate the digestive process of SWP, allowing it to be digested and hydrolyzed into smaller units, such as proteins or peptides. On the one hand, this may be because the physical effect of sonication blunts the activity of trypsin inhibitors; on the other hand, it may be that sonication unfolds the structure of SWP, thus exposing more sites for interaction with digestive enzymes [58]. The incorporation of Que also significantly improved the digestibility of SWP, with the particle size of the SWP–Que digestion product being only 241.90 ± 6.62 nm. This may be due to inactivation of trypsin activity [59]. In addition, the utilization of ultrasound treatment led to a notable reduction in particle size of the composite nanoparticles digestion product, which indicated a synergistic effect between ultrasound treatment and Que in blunting trypsin inhibitor activity.

#### 3.11.2 Que bioaccessibility analysis

After simulated gastrointestinal digestion, Que present in the supernatant of the digestive fluid is considered to be the fraction that can be absorbed by the intestinal mucosa, i.e., bioaccessible [31]. Fig. 6**E** shows that the bioaccessibility of Que in the composite nanoparticles was markedly higher than that of free Que (27.86 ± 2.07 %), displaying a pattern of initial increase followed by decline with rising ultrasound power. This may be due to the binding of Que to SWP, which occupies part of the site where digestive enzymes bind to SWP [60]. With the ultrasound power increased, the structure of SWP further unfolds, enhancing its binds to Que. When the ultrasound power was 400 W, the bioaccessibility of Que was the highest, reaching 64.32 ± 1.95 %. Nevertheless, when the treatment power was excessive, the depolymerised proteins were re–polymerized which also led to the decrease of Que binding. These results suggest that the binding of SWP to Que can effectively delay the degradation of Que during digestion, thereby improving its bioaccessibility.

## 4 Conclusion

In this experiment, SWP–Que self–assembled nanoparticles were successfully prepared, with the main driving force being hydrophobic interactions. Compared with natural SWP, ultrasound treated SWP has a more flexible secondary structure and looser tertiary conformation. The unfolding of its structure exposes more hydrophobic groups inside, which provides more binding sites for Que. In addition, the formation of nanoparticles also provided a protective effect for Que, which in turn slowed down its degradation under different environmental stresses (heat, UV light, and storage). In vitro simulated digestion experiments showed increased bioavailability of Que in encapsulated nanoparticles, which contributed to a better utilisation of Que for its biological activities. However, their ability to maintain excellent properties after being added to products for actual production, as well as bioavailability and bioactivity *in vivo* after consumption need to be further investigated. The present study provides a preliminary basis for expanding the application areas of SWP and the effective utilisation of flavonoids.

## CRediT authorship contribution statement

**Xinru Cao:** Writing – original draft, Investigation, Conceptualization. **Jia Cao:** Investigation, Data curation, Conceptualization. **Tianhe Xu:** Writing – review & editing. **Lexi Zheng:** Investigation. **Jingyi Dai:** Software. **Xiaokun Zhang:** Investigation. **Tian Tian:** Data curation. **Kunyu Ren:** Visualization. **Xiaohong Tong:** Resources, Methodology, Conceptualization. **Huan Wang:** Writing – review & editing, Supervision, Funding acquisition, Conceptualization. **Lianzhou Jiang:** Supervision.

## Declaration of competing interest

The authors declare that they have no known competing financial interests or personal relationships that could have appeared to influence the work reported in this paper.
